# The validation of Short Interspersed Nuclear Elements (SINEs) as a RT-qPCR normalization strategy in a rodent model for temporal lobe epilepsy

**DOI:** 10.1371/journal.pone.0210567

**Published:** 2019-01-10

**Authors:** René A. J. Crans, Jana Janssens, Sofie Daelemans, Elise Wouters, Robrecht Raedt, Debby Van Dam, Peter P. De Deyn, Kathleen Van Craenenbroeck, Christophe P. Stove

**Affiliations:** 1 Laboratory for GPCR Expression and Signal Transduction (L-GEST) - Laboratory of Toxicology, Department of Bioanalysis, Ghent University, Ghent, Belgium; 2 Laboratory of Neurochemistry and Behavior, Department of Biomedical Sciences, Institute Born-Bunge, University of Antwerp, Antwerp, Belgium; 3 Laboratory for Clinical and Experimental Neurophysiology, Neurobiology and Neuropsychology (LCEN3), Department of Internal Medicine, Ghent University Hospital, Ghent, Belgium; 4 Department of Neurology and Alzheimer Research Center, University Medical Center Groningen (UMCG), Groningen, the Netherlands; 5 Department of Neurology and Memory Clinic, Hospital Network Antwerp (ZNA) Middelheim and Hoge Beuken, Antwerp, Belgium; 6 Biobank, Institute Born-Bunge, University of Antwerp, Antwerp, Belgium; Northwestern University Feinberg School of Medicine, UNITED STATES

## Abstract

**Background:**

In gene expression studies via RT-qPCR many conclusions are inferred by using reference genes. However, it is generally known that also reference genes could be differentially expressed between various tissue types, experimental conditions and animal models. An increasing amount of studies have been performed to validate the stability of reference genes. In this study, two rodent-specific Short Interspersed Nuclear Elements (SINEs), which are located throughout the transcriptome, were validated and assessed against nine reference genes in a model of Temporal Lobe Epilepsy (TLE). Two different brain regions (i.e. hippocampus and cortex) and two different disease stages (i.e. acute phase and chronic phase) of the systemic kainic acid rat model for TLE were analyzed by performing expression analyses with the geNorm and NormFinder algorithms. Finally, we performed a rank aggregation analysis and validated the reference genes and the rodent-specific SINEs (i.e. B elements) individually via *Gfap* gene expression.

**Results:**

GeNorm ranked *Hprt1*, *Pgk1* and *Ywhaz* as the most stable genes in the acute phase, while *Gusb* and *B2m* were ranked as the most unstable, being significantly upregulated. The two B elements were ranked as most stable for both brain regions in the chronic phase by geNorm. In contrast, NormFinder ranked the B1 element only once as second best in cortical tissue for the chronic phase. Interestingly, using only one of the two algorithms would have led to skewed conclusions. Finally, the rank aggregation method indicated the use of the B1 element as the best option to normalize target genes, independent of the disease progression and brain region. This result was supported by the expression profile of *Gfap*.

**Conclusion:**

In this study, we demonstrate the potential of implementing SINEs -notably the B1 element- as a stable normalization factor in a rodent model of TLE, independent of brain region or disease progression.

## Introduction

The reverse transcription quantitative polymerase chain reaction (RT-qPCR) is considered as the most sensitive, reliable and accurate technique to analyze differential gene expression at the messenger RNA (mRNA) level [1, 2]. Nevertheless, performing the experimental procedures will introduce a variety of potential errors, amongst which pipetting errors, starting material quality variations, differences in mRNA extractions, errors in sample quantifications, altered reverse transcription efficiencies and cDNA sample loading differences [3]. In order to correct for these variations, a normalization strategy is required (e.g. starting with similar amounts of cells or input mRNA). The most popular strategy is to use one or multiple endogenously expressed control genes (i.e. reference genes), such as ribosomal RNAs (rRNA) or mRNAs [4]. Ideally, a reference gene should be abundantly expressed, not co-regulated with the target gene and have a similar expression across all samples [5]. Many RT-qPCR studies implement reference genes that are related to structural or basic processes (e.g. *Actb* or *Gapdh*). However, several reference genes have been shown to be differentially expressed between samples, cell types and experimental conditions [6–8]. In recent years, an increasing amount of studies have validated and assessed the stability of those genes in specific pathological models [9–11]. A reference gene in one animal model may be differentially expressed, while the same gene will be stably expressed in another model of the same pathology. Also, the reference gene expression in different tissues of the same model may vary [12–14].

Several research groups reported a new normalization strategy with Expressed Alu Repeats (EARs) and Expressed Repeat Elements (EREs) as internal control genes, in *Homo sapiens* and *Danio rerio*, respectively [15–18]. Those studies proposed to use these repeat elements, which are located throughout the transcriptome, as a normalization factor in RT-qPCR analysis. The EARs or EREs would be less influenced by the up- or downregulation of one or more transcripts, which would make the normalization process more reliable over various experimental conditions, compared to the use of a single reference gene. This would reduce the workload and cost in reference gene validation and avoid the loss of valuable biological materials [15–18].

The rodent genome (e.g. from *Rattus norvegicus*) does not contain Alu repeats, but B1, B2, ID and B4 elements (i.e. Alu-like elements) instead, which belong to the class of Short Interspersed Nuclear Elements (SINEs) [19]. The Alu repeats and the B1 elements share their origin from an initial duplication of the 7SL RNA before the primate-rodent split, 80 million years ago. These two sequences have been amplified and duplicated independently with accumulating mutations and have little resemblance to each other or to the original 7SL RNA. The 7SL RNA-derived SINEs are unique in the genomes of primates, rodents and tree-shrews [20–23]. The B2 element is rodent-specific and has a tRNA-like region with an unknown affiliation, combined with a unique 120 bp sequence. A neuronally expressed BC1 gene is believed to be the origin of the ID elements [24]. In contrast, the B4 element resembles a fusion of a B1 and an ID element. The B elements, as well as the Alu repeats, are dispersed throughout the transcriptome, with the highest density in the intronic regions and at 16 kb upstream to the start position of specific protein-coding transcripts. In contrast, they are under-represented in exonic regions [25–27].

Previous RT-qPCR studies tried to obtain a better understanding of the molecular mechanisms of epilepsy, without or with a limited reference gene validation study [28–31]. In the present study, we have evaluated the stability of nine well-accepted reference genes and two SINEs (B1 and B2 elements) in the systemic kainic acid (KA) post status epilepticus (SE) rat model of Temporal Lobe Epilepsy (TLE). The expression stability of the transcripts was determined in the hippocampus and the cortex during the acute phase (day 10 post-SE) and chronic phase (day 80 and 120 post-SE) of disease progression (i.e. epileptogenesis). The RT-qPCR results were analyzed with geNorm [32], NormFinder [33] and rank aggregation [34] algorithms. As a validation strategy, normalization using the geNorm and NormFinder results was compared to normalization using the B elements or every single reference gene in the context of evaluation of the relative expression of glial fibrillary acidic protein (*Gfap*) mRNA. To our knowledge, this is the first large-scale RT-qPCR study which validated the robustness of B elements in a rodent model.

## Materials and methods

### Animals

Male Sprague-Dawley rats (Harlan Laboratories B.V., the Netherlands) weighing 242.6 ± 15.1 g (9 weeks) were treated according to the guidelines of the European Communities Council Directive (2010/63/EU). The Animal Experimental Ethical Committee of Ghent University Hospital (ECD 16/06) approved the study protocol. The animals were conventionally housed in a temperature-controlled (20–23°C) and humidity-controlled (50%) environment under a 12h/12h light/dark cycle, where food and water intake was *ad libitum*.

The rats were randomly distributed for KA administration (n = 19) and controls (n = 12). In order to induce SE, rats received 2–6 times KA (5 mg/kg; Tocris Bioscience, Bristol, UK) by intraperitoneal (i.p.) injections according to the protocol of Hellier et al., 1998. Seizure activity of all rats was continuously monitored visually and electrographically. The KA treatment was repeated hourly until the animals displayed a stable self-sustained SE for ≥3 h (i.e., >10 behavioral seizures per hour). Animals that exhibited excessive motor or excessive lethargic behavior were no longer injected with KA to avoid mortality [35, 36]. The rats treated with KA were sampled after 10 (T10, n = 6), 80 (T80, n = 7) or 120 days (T120, n = 6). The control rats were treated with vehicle (saline, i.p.) and were sampled after 10 (acute phase control, n = 6) or 120 days (chronic phase control, n = 6). The animals were anesthetized via 5% isoflurane (Affygility Solutions, Broomfield, CO, USA) followed by decapitation and hippocampi and cortices were dissected on an ice-cold plate, the left and right parts were stored separately at -80°C until RNA isolation.

### RNA extraction and cDNA synthesis

Total RNA was isolated from left hippocampi and cortices with the RNeasy Plus Universal Mini Kit (Qiagen, Hilden, Germany), according to the manufacturer’s instructions. The absorbance at 230 nm, 260 nm, 280 nm and total RNA concentration were measured with the NanoDrop 1000 Spectrophotometer (ThermoFisher Scientific, Wilmington, DE, USA). The 260/280 nm ratio was used as RNA purity measure and all samples had values over 2.0. The integrity of the RNA was assessed by the analysis of the ratio of 28S to 18S rRNAs after agarose gel electrophoresis. Total RNA samples (15 μg) were treated with the Heat&Run gDNA removal kit (ArticZymes, Tromsø, Norway) to avoid amplification of genomic DNA during the reverse transcription.

The concentrations and purities were verified again, as described above. Only 1.5 μg of the total RNA was converted to cDNA using Oligo(dT)18 primers (Cell Signaling, Danvers, MA, USA) and M-MLV Reverse Transcriptase (Promega, Madison, WI, USA), according to the manufacturer’s protocol. The absence of DNA contamination was controlled for each individual sample by omitting the reverse transcriptase (RT) from the procedure (i.e. minus RT samples). The minus RT and the plus RT samples of the control and the KA-treated samples of a similar disease progression stage (i.e. acute or chronic phase) were reverse transcribed simultaneously. After 1:20 dilution of the samples, a 10 μl fraction of every cDNA was pooled together in order to determine the PCR efficiency for every primer set. All samples were stored at -80°C until further analysis.

### Primer design and real-time PCR

Nine commonly used reference genes were chosen based on their differences in cellular or signaling pathways (Table 1). The primers for the nine reference genes and *Gfap* were found in literature [9, 14]. The primers for the B1 and B2 elements were designed based on the available consensus sequences [20, 37]. The optimal primers were chosen from the regions with the highest consensus sequences and specificity was controlled *in silico* through the UCSC genome browser (Santa Cruz, CA, USA). All primers were synthesized by Sigma Aldrich (St. Louis, MO, USA).

**Table 1:** Name and function of genes and short interspersed nuclear elements.

Symbol	Gene name	Function
Actb	Actin beta	Cytoskeletal structural protein
B1 element	B1 short interspersed nuclear element	Wide-spread class or repeat element through the mammalian genome and descended from 7SL RNA
B2 element	B2 short interspersed nuclear element	Wide-spread class or repeat element through the mammalian genome with a tRNA-like region followed by a unique 120 bp region
B2m	Beta-2 microglobulin	Beta-chain of major histocompatibility complex class I molecules
Gapdh	Glyceraldehyde-3-phosphate dehydrogenase	Glycolytic enzyme
Gusb	Glucuronidase beta	Hydrolyzes and degrades glycosaminoglycans
Hprt1	Hypoxanthine guanine phosphoribosyl transferase 1	Purine synthesis in salvage pathway
Pgk1	Phosphoglycerate kinase I	Glycolytic enzyme
Rpl13a	Ribosomal protein L13A	Structural component of the large 60S ribosomal subunit
Tbp	TATA box binding protein	General RNA polymerase II transcription factor
Ywhaz	Tyrosine 3-monooxygenase/tryptophan 5-monooxygenase activation protein, zeta polypeptide	Signal transduction by binding to phosphorylated serine residues on a variety of signaling molecules

Real-time PCRs were performed in white 96-well plates using the LightCycler 480 Instrument (Roche Diagnostics, Mannheim, Germany). In a reaction volume of 15 μl, 5 μl of the synthesized cDNA sample was pipetted. Thereby, the synthesized cDNA was diluted 1:40 for the reference genes and *Gfap* and 1:8000 for the B elements, to 3.75 ng and 0.019 ng of total cDNA respectively. The primer concentrations were 250 nM. Finally, 7.5 μl iQ SYBR Green Supermix (Bio-Rad Laboratories, Hercules, CA, USA) and 1.75 μl DEPC H_2_O was added to the reaction volume. The two-step amplification protocol of the real-time PCRs was as follows: initial denaturation at 95°C for 5 minutes and 40 cycles of 95°C for 15 seconds and 60°C for 45 seconds. A light signal was acquired at the end of each cycle at 60°C. The specificity of product formation was confirmed by a melting curve analysis (55°C to 95°C in increments of 0.11°C/s). The Cq values were determined in technical triplicates with the LightCycler software v1.1.5 (Roche Applied Science, Mannheim, Germany) according to the second derivative method.

The PCR efficiencies were determined by technical duplications of a three- or five-point serial dilution of pooled cDNA, B elements or reference genes respectively. All the PCR efficiencies of the primer sets were between 90 and 110% (Table 2), demonstrating the robustness and reproducibility of the performed RT-qPCR assay.

**Table 2:** Primer sequences and amplicon characteristics for the *Rattus norvegicus* used in the study.

Symbol	5’-3’ sequence	Reference	Amplicon Length (bp)	PCR efficiency (%)
Actb	AGCCTTCCTTCCTGGGTATG GAGGTCTTTACGGATGTCAAC	NM_031144.2	92	91.1
B1 Element	ACGCCTTTAATCCCAGCACTC GAACTCACTCTGTAGACCAGGCTG	Veniaminove et al., 2007	81–83	98.9
B2 Element	CCACATGGTGGCTCACAAC CCAGAAGAGGGCATCAGATC	Dunnen et al., 1987	51–64	98.7
B2m	CGAGACCGATGTATATGCTTGC GTCCAGATGATTCAGAGCTCCA	NM_012512	118	92.5
Gapdh	CCCATTCTTCCACCTTTGATGCT CTGTTGCTGTAGCCATATTCAT	NM_017008.3	104	95.0
Gfap	AACCGCATCACCATTCCTGT CATCTCCACCGTCTTTACCAC	NM_017009.2	123	97.7
Gusb	CCGTGGAACAGGGAATGAG CTCAGGTGTTGTCATCGTCA	NM_017015.2	121	98.9
Hprt1	CTCATGGACTGATTATGGACAGGAC GCAGGTCAGCAAAGAACTTATAGCC	NM_012583	123	92.4
Pgk1	ATGCAAAGACTGGCCAAGCTAC AGCCACAGCCTCAGCATATTTC	NM_053291	104	95.8
Rp113a	GGATCCCTCCACCCTATGACA CTGGTACTTCCACCCGACCTC	NM_173340	132	95.2
Tbp	TGGGATTGTACCACAGCTCCA CTCATGATGACTGCAGCAAACC	NM_001004198	131	90.3
Ywhaz	GATGAAGCCATTGCTGAACTTG GTCTCCTTGGGTATCCGATGTC	NM_013011	117	92.9

### Gene expression stability analysis and reference gene validation

The stability of the reference genes and B elements was assessed by geNorm (https://genorm.cmgg.be) and NormFinder (https://moma.dk/normfinder-software) [32, 33]. In order to perform the geNorm analysis, the Cq values were imported into the qBaseplus software version 3.0 (Biogazelle, Ghent, Belgium) [38]. This algorithm calculates the gene stability measure M, which is the average pairwise variation of a particular gene with all other genes. The genes are ranked according to the calculated M value from the least stable (highest M value) to the most stable (lowest M value). The NormFinder algorithm estimates the stability through a model-based approach by comparing the overall gene expression variation and the gene variation between sample subgroups. This results in a stability value and ranks the genes based on their stable expression. The Cq values used in both algorithms were efficiency adjusted.

All the ranked gene lists obtained by the geNorm and NormFinder analyses were used in the rank aggregation R package called “RankAggreg” [34]. The weighted rank aggregation was applied to determine the most stable reference genes across the different disease stages, brain regions and algorithms. Thereby, we used the Cross-Entropy (CE) Monte Carlo algorithm, which generates random lists. The starting list was converged towards the best optimal list through an iteration procedure with the weighted Spearman’s foot-rule distance, resulting in a consensus list of ranks.

According to literature, patients and different animal models of TLE show significant upregulation in *Gfap* mRNA expressions [10, 14, 39–41]. For that reason, *Gfap* mRNA expression was chosen to evaluate the impact of the different kind of normalization strategies. The *Gfap* expression was evaluated with the most stable combination of references genes according to the two algorithms or to the eleven references independently, using the 2^ΔΔ*CT*^ method [38].

The Cq values of each individual reference gene and the *Gfap* expression values, which were determined by different normalization methods, in the hippocampus and cortex were imported into GraphPad Prism version 6.00 for Windows (GraphPad Software, La Jolla, CA, USA). The various groups were statistically analyzed with the Mann-Whitney two-tailed U test (i.e. acute phase) or the nonparametric ANOVA by ranks of Kruskal-Wallis test followed by the Dunn’s multiple comparisons post-hoc test (i.e. chronic phase). Differences were considered statistically significant at P ≤ 0.05.

## Results

### Transcription profiles

**Fig 1:**
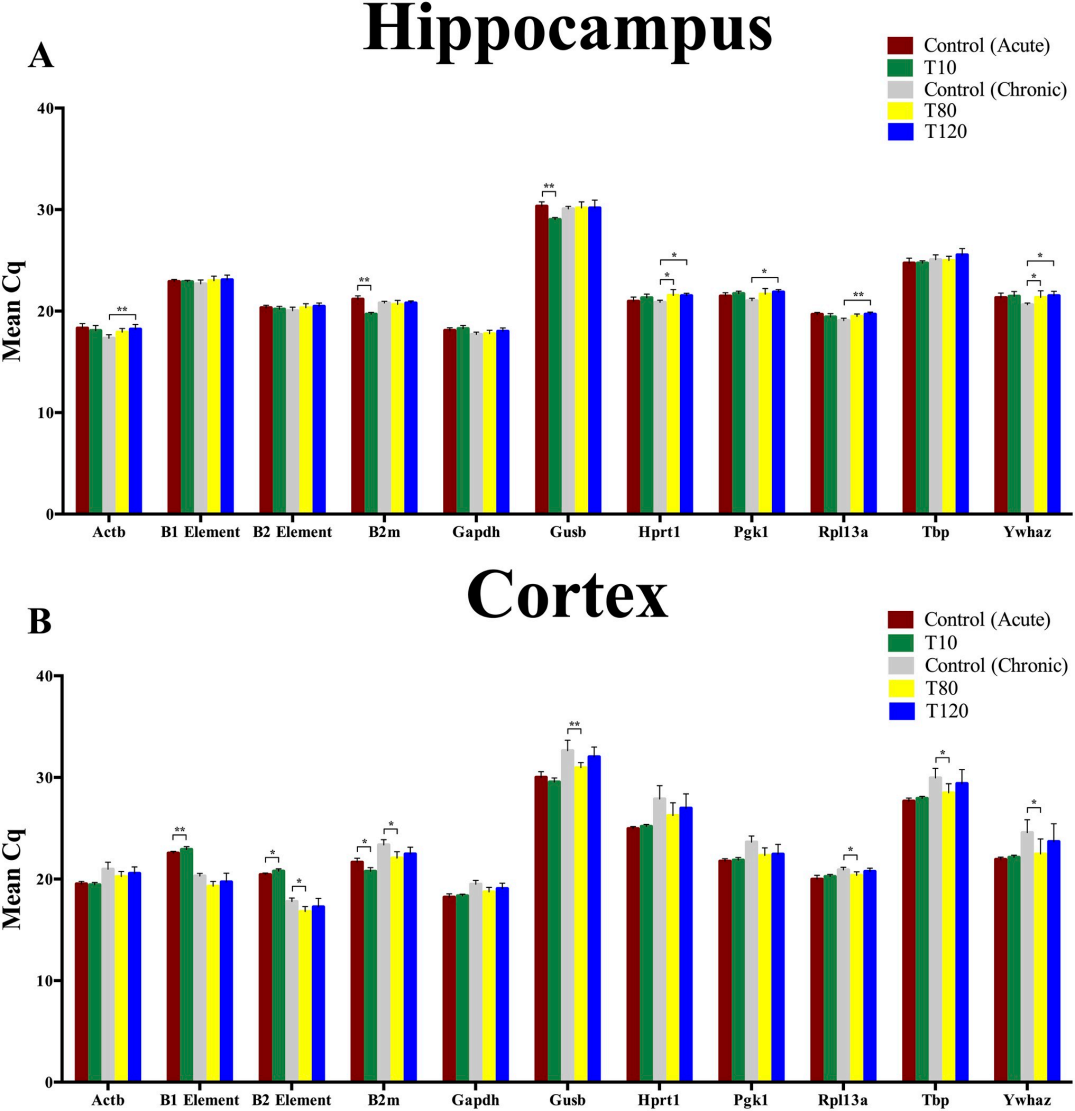
Cq values of the reference genes and B elements in hippocampus and cortex. (A) The mRNA expression profiles of the reference genes and B elements in the hippocampus of the acute phase (T10), the chronic phase (T80 and T120) in the KA model and their corresponding controls. (B) The mRNA expression profiles of the reference genes and B elements in the cortex of the acute phase (T10), the chronic phase (T80 and T120) in the KA model and their corresponding controls. Results are given in Cq values, mean ± SD (n = 6/7), during the acute phase (acute phase control vs. T10) and chronic phase (chronic phase control vs. T80/T120) phase, Mann-Whitney U Test or Kruskal-Wallis Test, respectively, * = P ≤ 0.05 and ** P ≤ 0.01.

The mean Cq values represent a different intragroup variation profile of the reference genes in the hippocampus compared to the cortex (Fig 1). The genes *B2m* and *Gusb* had a significantly higher expression in the TLE model than in the controls at the acute phase (acute phase control vs. T10), with the exception of the *Gusb* cortical expression. The chronic phase shows a more disperse pattern, where the mRNA expression of *Hprt1*, *Pgk1*, *Ywhaz*, *Actb* and *Rpl13a* differs significantly between the control and TLE rats in the hippocampus, while *Gusb*, *B2m*, *Rpl13a*, *Tbp*, *Ywhaz* and the B2 element differ significantly between groups in the cortex. The significant differences in mean Cq values may indicate the instability of a reference gene or B element. The Cq value profile displayed a wide range of mRNA expression levels, where *Gusb* has high Cq values (30.42), thus a low mRNA expression, and *Gapdh* (18.37), *Actb* (19.13) and B2 element (19.41) were highly expressed.

### Stability determination

**Fig 2:**
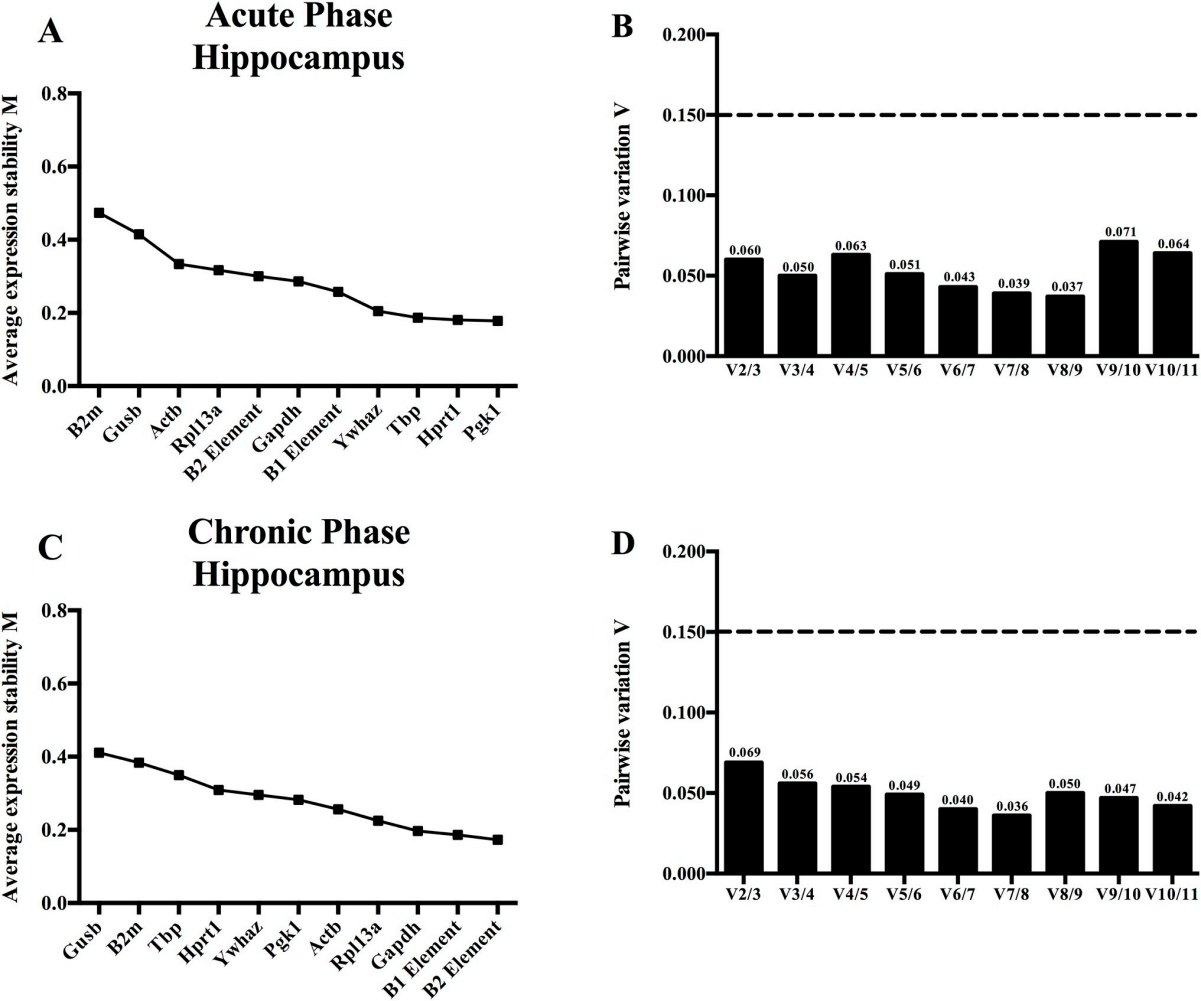
A geNorm analysis of the acute and chronic phase in the hippocampus of the TLE model. (A) Expression stability values (M values) of all the reference genes and B elements in the hippocampus of the acute phase. The higher the M value, the less stable the gene is expressed and vice versa. (B) Pair-wise variation analysis to determine the number of optimal genes for normalization in the acute phase. The software calculates a V value, which is an expressed variation number between the two calculated sequential normalization factors. (C) The M values of all the reference genes and B elements in the hippocampus of the chronic phase. (D) Pair-wise variation analysis to determine the number of optimal genes for normalization in the chronic phase.

The expression stability of the reference genes and the B elements was assessed by two algorithms. The geNorm algorithm labels the most stable genes with the lowest M value. This value is defined as the mean standard deviation of the logarithmically transformed expression values of the compared genes, which is calculated by the average variation among pairs of genes through the comparison of a control gene with other genes. First, the algorithm selects a pair of genes, which have the lowest M value, the additional genes are ranked based on the highest degree of compatibility with the other and with a geometric mean of the first pair [38]. The order of stability of the reference genes and B elements varied between the acute and chronic phase in the hippocampus (Fig 2A and 2C).

Nowadays, to perform correct normalization of a target gene (e.g. *Gfap*), multiple reference genes are recommended [4]. The qBaseplus software package also performs a pairwise variation analysis. This analysis calculates the variation between successive pairs, which is expressed as the normalization factor (NF). Hereby, the influence of a subsequent reference gene added to the previous pair is determined. The comparison is made with NF_n_ for n number of genes to NF_n+1_, which contain the same set of genes with an additional gene having a consecutive higher M value. If the added gene to the NF has a significant impact, the variation V_n/n+1_ between two NF factors is high. Supported by the data presented in the original publication, a cutoff value of 0.15 for V_n/n+1_ was determined [32]. When the value is below the cutoff, the addition of an extra reference gene is not necessary for the normalization of a target gene. Using two reference genes in the acute (*Pgk1*/*Hprt1*) or chronic (B1 and B2 element) model is sufficient, with values of 0.060 and 0.069 respectively (Fig 2B and 2D).

**Fig 3:**
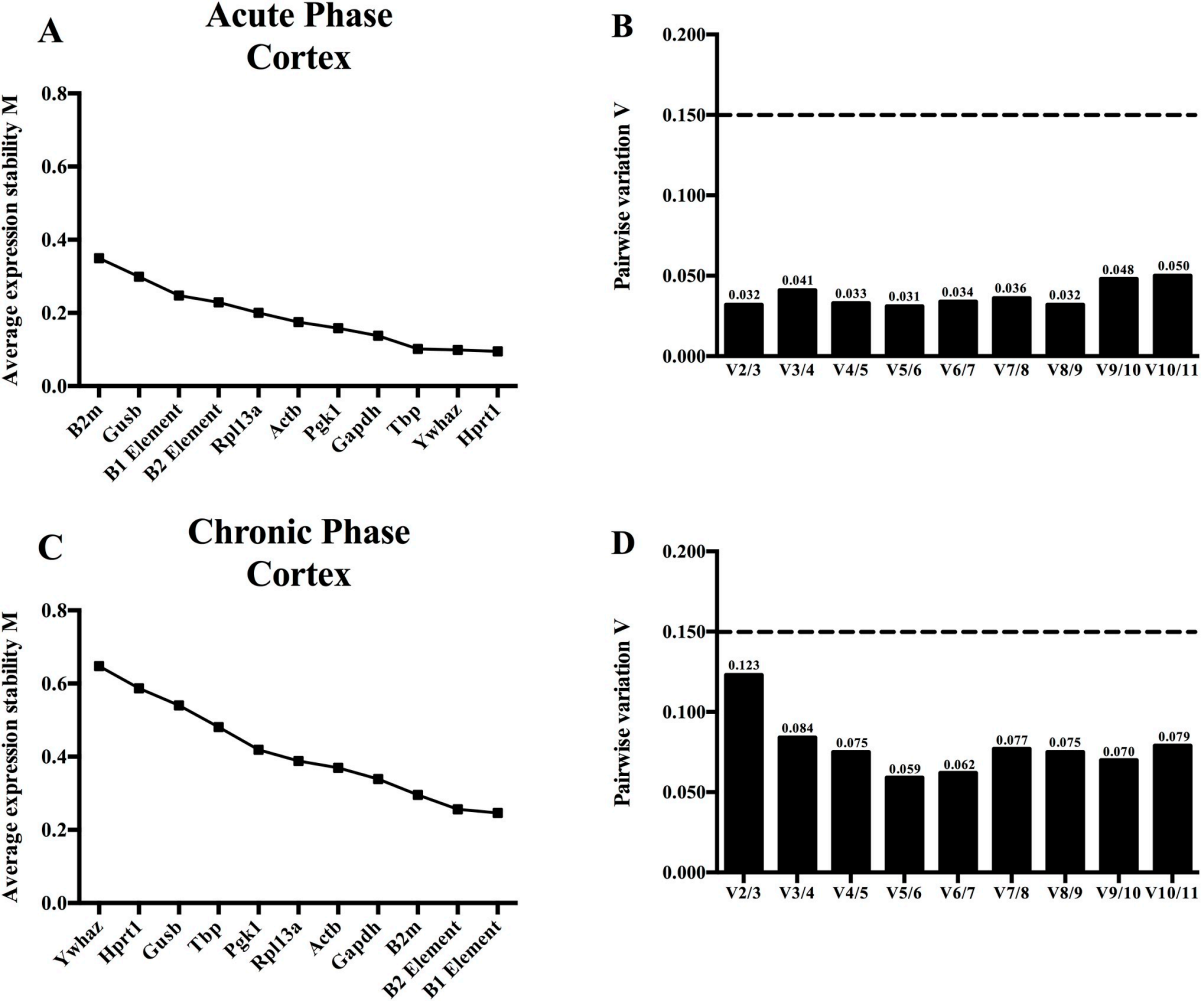
A geNorm analysis of the acute and chronic phase in the cortex of the TLE model. (A) Expression stability values (M values) of all the reference genes and B elements in the cortex of the acute phase. The higher the M value, the less stable the gene is expressed and vice versa. (B) Pair-wise variation analysis to determine the number of optimal genes for normalization in the acute phase. The software calculates a V value, which is an expressed variation number between the two calculated sequential normalization factors. (C) The M values of all the reference genes and B elements in the cortex of the chronic phase. (D) Pair-wise variation analysis to determine the number of optimal genes for normalization in the chronic phase.

An equivalent geNorm analysis was performed in the cortex of the rat model for TLE (Fig 3). The algorithm determined that the *Hprt1* and *Ywhaz* reference genes were sufficient for the normalization (V_2/3_ = 0.032) in the acute phase. Similar as in the hippocampus, the B elements were also the most stably expressed in the chronic phase. The V value (0.123) is below the defined cutoff value (0.15), so according to the geNorm algorithm, the use of only the two B elements as reference genes is adequate for normalization.

The NormFinder algorithm uses a model-based estimation of variance approach to determine a stability value. This ANOVA model-based approach takes into account the average influence of a gene within the group and the individual impact of a group compared to the other groups, called the intra- and intergroup variations, respectively. The final value depends on the number of candidate genes and samples analyzed [33]. The obtained results of the NormFinder analysis of this study are shown in Table 3. In the acute phase, the two most stable genes in the hippocampus are *Rpl13a* (0.164) and *Actb* (0.185), while *Gusb* (0.611) and *B2m* (0.693) are the two most unstable genes. In the chronic phase the three most stable genes were *Rpl13a* (0.129), *Gapdh* (0.143) and the B1 element (0.159). The most unstable genes were *B2m* (0.314) and *Tbp* (0.345), which differed slightly from the geNorm analysis. In the acute phase, the most stable reference genes in the cortex are *Actb* (0.112) and *Gapdh* (0.145). As before, the two most unstable genes were *Gusb* (0.356) and *B2m* (0.508). In the chronic phase, *B2m* (0.196) and B1 element (0.240) have the least diversity, while *Rpl13a* (0.359) and *Ywhaz* (0.437) turn out to be unreliable as reference genes. The calculated intra- and intergroup variations per gene are presented in S1 Table.

**Table 3:** A NormFinder analysis of the acute (acute phase control vs. T10) and the chronic (chronic phase control vs. T80/T120) phase in the hippocampus and cortex.

Gene name	Acute Phase Hippocampus	Chronic Phase Hippocampus	Acute Phase Cortex	Chronic Phase Cortex
Actb	***0*.*185***	0.229	***0*.*112***	0.266
B1 Element	0.231	0.159	0.233	***0*.*240***
B2 Element	0.193	0.298	0.228	0.264
B2m	0.693	0.314	0.508	***0*.*196***
Gapdh	0.334	***0*.*143***	***0*.*145***	0.261
Gusb	0.611	0.274	0.356	0.331
Hprt1	0.381	0.211	0.163	0.333
Pgk1	0.278	0.257	0.159	0.261
Rpl13a	***0*.*164***	***0*.*129***	0.216	0.359
Tbp	0.190	0.345	0.208	0.251
Ywhaz	0.337	0.280	0.168	0.437
Best Genes	Rpl13a & Actb	Rpl13a & Gapdh	Actb & Gapdh	B2m & B1 Element

### Rank aggregation

**Fig 4:**
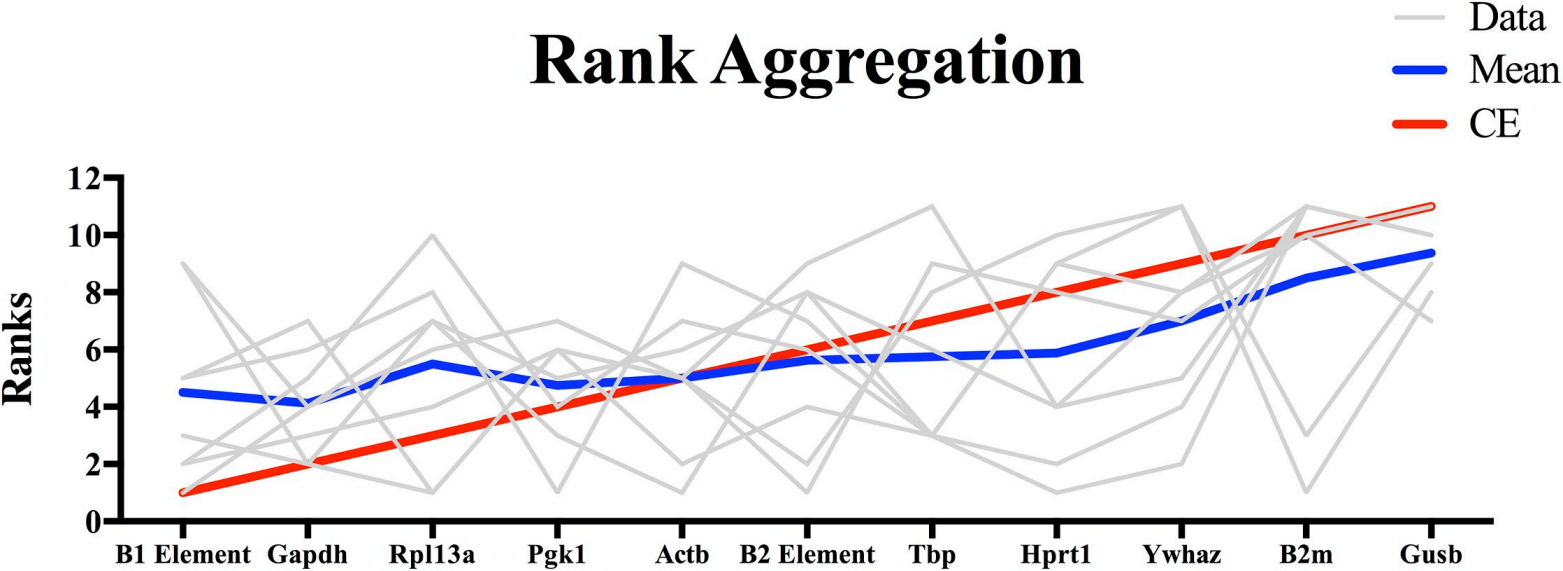
The rank aggregation consensus result of the reference genes and B elements via the performance of the Cross-Entropy (CE) Monte Carlo algorithm in combination with the Spearman’s foot-rule weighted distance.

Not surprisingly, the different algorithms yielded a different ranking of the genes and B elements. In order to obtain a final consensus list of ranks, weighted rank aggregation was conducted. The R script for the weighted rank aggregation, via a CE Monte Carlo algorithm with the weighted Spearman’s foot-rule distance, is presented in S1 File. The weight of the M values of the geNorm algorithm and the stability values of the NormFinder algorithm were taken into account in this iterative process [34]. As presented in Fig 4, the B1 element is the most stably expressed, independent of brain region and disease stage.

### Validation of reference genes and B elements

**Fig 5:**
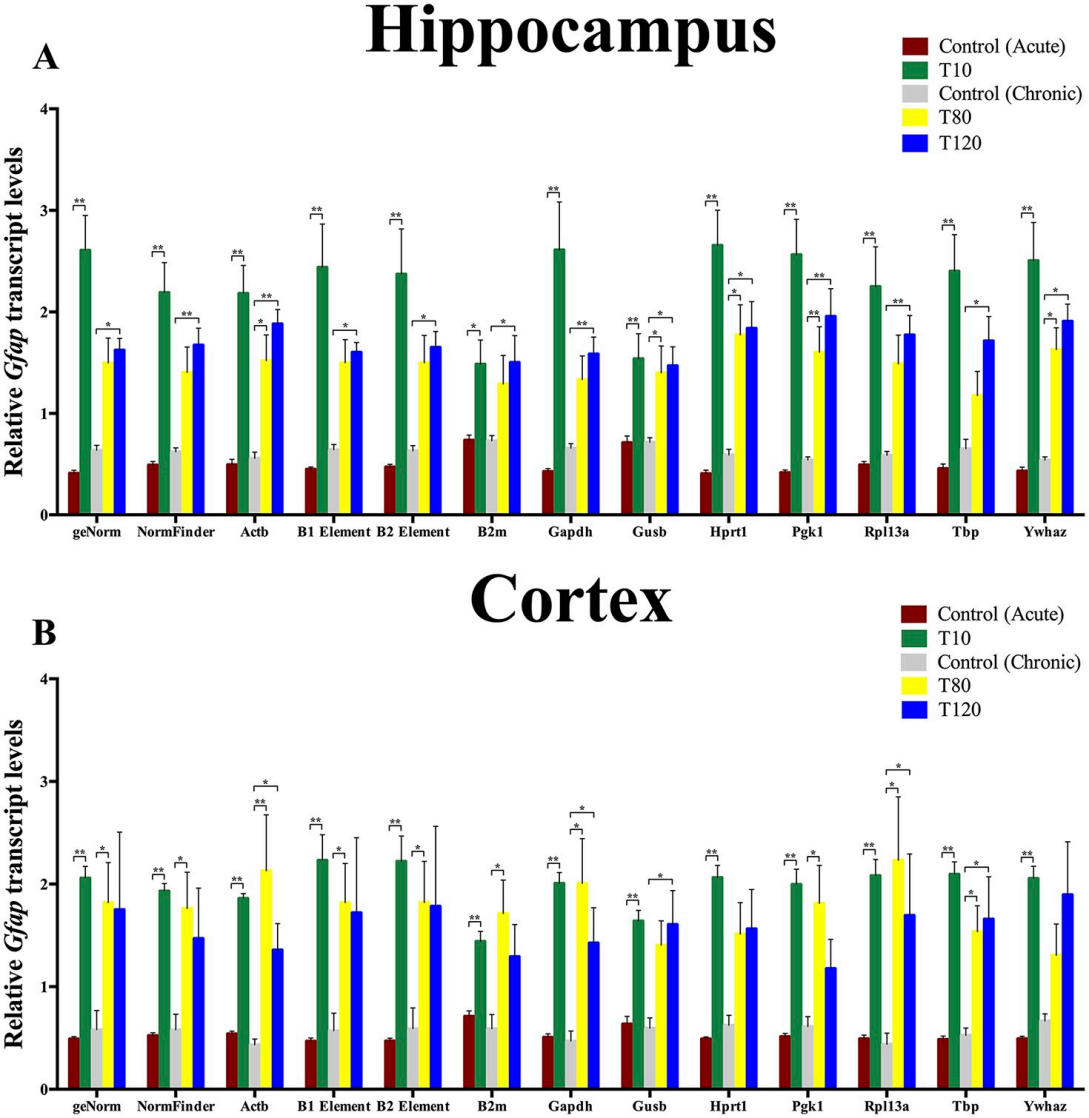
Transcription profiles of *Gfap* in two brain regions upon using different normalization approaches. (A) The relative expression of *Gfap* in the hippocampus, normalized using each of the nine reference genes, the B elements or the best combination derived from the NormFinder and geNorm analysis. (B) The relative expression of *Gfap* in the cortex normalized by nine reference genes, B elements or the best combination derived from the NormFinder and geNorm analysis. The graphs show the mean *Gfap* expression during the acute (acute phase control vs. T10) and chronic (chronic phase control vs. T80/T120) phase, Mann-Whitney U Test or Kruskal-Wallis Test respectively, * = P ≤ 0.05, ** = P ≤ 0.01.

Finally, the B elements and reference genes used in this study were validated to normalize the expression analysis of *Gfap*. From literature, it is known that expression of this gene is upregulated in several epilepsy models as well as in human patients with TLE [10, 14, 39–41]. The best pairs of genes resulting from the geNorm and NormFinder analysis and all the individually expressed genes and B elements were used to evaluate the pattern of *Gfap* in the hippocampus and the cortex, as depicted in Fig 5A and 5B, respectively. Normalized *Gfap* expression was very similar for both B elements as reference genes, in the hippocampus, as well as in the cortex. Moreover, normalization using the B elements was almost equivalent to normalization using the geNorm and NormFinder method. Only the chronic phase control versus the T120 in the NormFinder differed slightly compared to the B1 or B2 element normalization in the hippocampus. Even the use of unstably expressed genes as *Gusb* and *B2m* still allowed detection of the significant upregulation of *Gfap* mRNA expression in the two brain regions, in both the acute and chronic phase. Nevertheless, the upregulation of *Gfap* normalized by these reference genes differed in significance level compared to the normalization of *Gfap* with geNorm and NormFinder. Moreover, these reference genes were significantly upregulated in the acute phase of the TLE model, independent of brain region (S1 Fig). Remarkably, in the chronic phase of the TLE model normalization using *Ywhaz* didn’t yield significant *Gfap* upregulation in the cortex.

## Discussion

Currently, RT-qPCR is the ‘gold standard’ to determine variation in mRNA expression levels in biological materials between experimental conditions. Hereby, an accurate normalization strategy is key to infer correct conclusions from generated data. Predominantly, the expression of one or more endogenous ‘reference’ genes is used to correct for technical variations in the RT-qPCR protocol. Using multiple reference genes is recommended and the optimal number applied should be experimentally determined [4]. Importantly, some experimental procedures also influence reference gene expression, rendering specific genes unsuitable for normalization of target gene expression. Applying only a single reference gene could lead to erroneous conclusions in that case [32].

Our study is the first to validate the use of SINEs as a normalization strategy, comparing these to nine widely used and accepted reference genes as a normalization method in a rodent model. Previously, SINEs were validated in zebrafish tissues, human blood samples and human cell lines [15–18]. In order to perform the comparison, we selected nine reference genes from different functional classes, for example genes involved in major histocompatibility complex (*B2m*), transcription (*Tbp*) or metabolism (*Gapdh*), to avoid co-regulation (Table 1). The use of ribosomal RNA (rRNA) as a normalizer is discouraged, because the rRNA (e.g. 18S rRNA) is not polyadenylated, transcribed by a different RNA polymerase and has a different function in the cell than mRNA. Although around 90% of the total RNA concentration contains rRNA, the mRNA/rRNA ratio can fluctuate depending on the experimental conditions [42, 43]. Therefore, the rRNA was not included in our research. In contrast, the SINEs (i.e. B elements in the *Rattus norvegicus*) are part of the mRNA and are dispersed abundantly throughout the transcriptome.

The implementation as a normalization factor in RT-qPCR of another class of retrotransposons, the Long Interspersed Nuclear Elements (LINE-1), is discouraged as well. A difference in transcription of these autonomous retrotransposons cannot only be induced by various chemical and biological stressors, but also has been observed in cancers, neurodegenerative disorders and autoimmune diseases [44–49]. This would be due to hypomethylation of CpG islands and chromatin rearrangements [50–52]. In addition, a qPCR analysis of human brain tissue showed a high variation in LINE-1 copy number expression between individuals, especially in the hippocampus, and differential expression between neurons [53, 54].

The majority of the B elements are either located in upstream regions of specific transcripts, or in their intronic regions [25–27]. Hence, PCR amplification of a specific repeat from a B element will multiply the same transcript of various genes. Predictably, the detection of many transcripts simultaneously will be less influenced by an unstably expressed gene. As a consequence, it can be expected that the detected signal will be less prone to variation during various biological conditions, than when a single gene is detected in RT-qPCR.

As a proof-of-concept we used a rodent model of TLE and sampled two different brain regions at various disease stages (i.e. acute phase and chronic phase). KA will induce SE, followed by a latency period, and finally spontaneous recurrent seizures occur [55, 56]. The glutamate analog, KA, is a neuroexcitotoxic agent that acts via kainate receptors. Different pathologic changes will be manifested in the rodent, such as behavioral changes (e.g. occurrence of wet-dog shakes), electrophysiological alterations, induction of oxidative stress, astrogliosis and aberrant mossy fiber sprouting. Primarily, the CA3 region of the hippocampus has been shown as the most susceptible region. To a lesser extent the entorhinal- and piriform cortex are targeted by KA [57–59]. Several studies have proposed reference genes for intrahippocampal KA and pilocarpine (PILO) induced models of TLE and human epileptic brain tissue [10, 14, 60, 61]. Because the expression of a reference gene may be unstable in different (pathological) models, this study evaluated the suitability of nine specifically chosen reference genes, as well as B elements, to normalize gene expression in the rodent model of TLE via systemic KA administration [14]. Where most studies analyze only the hippocampus, we also included the cortex, which is known to be affected as well.

An extra added value of our analysis is the application of both the geNorm and NormFinder algorithm to determine the stability of the B elements, unlike previous studies, which validated SINEs and inferred their conclusions only from one algorithm (i.e. geNorm) [16–18]. As demonstrated by our data, there is a clear difference between the rankings generated by the two algorithms (Table 3, Figs 2 and 3). Where a target gene (e.g. *Gfap*) uses reference genes (e.g. *Gapdh*) as a point of reference to infer differences in expression over various experimental conditions, a reference gene itself does not have a point of reference. In order to select the most stable reference gene(s) over the experimental conditions, the two algorithms employ different mathematical schemes [32, 33]. To merge the results of both algorithms, we applied a rank aggregation strategy, resulting in one ranking scheme, independent of biological variables [34]. From this analysis, we could conclude that the B1 element, but not the B2 element, functions as the most stable reference in our hands. Others have also reported that not every repeat element is stably expressed under different conditions [17]. Upregulation of mouse B2 elements under heat shock conditions has been reported. Hereby, the B2 element binds to RNA polymerase II, acting as a transcriptional repressor of protein-coding genes to prevent translation, thus reducing the number of misfolded proteins under hyperthermia [62–64]. An opposite effect was seen in the hippocampus under stress conditions, wherein it was hypothesized that proteins should be translated in the hippocampus to retain memories of successful escapes and danger cues for future situations. The above demonstrates the ‘modulability’ of the B2 element, possibly explaining why in our model its expression was somewhat less stable than that of the B1 element [65].

Expression of the astrocyte-specific cytoskeleton protein *Gfap* was evaluated, relative to every single reference gene or B element independently, or relative to the results of geNorm or NormFinder. It has been well established for several models of TLE that *Gfap* expression is highly upregulated during astrogliosis in human and animal epileptogenic hippocampi [10, 14, 39–41]. The expression pattern, when normalizing with the B elements, was similar to that obtained when using geNorm or NormFinder, which is an indication of a specific upregulated pattern. Remarkably, *B2m* and *Gusb* were significantly upregulated in both brain regions of the acute phase. In a PILO model, when these two genes were used in normalization, a significant upregulation of *Gfap* expression was not detectable [14]. Clearly, the *Gusb* and *B2m* genes are unsuitable as references genes in the acute phase, since both were significantly upregulated (S1 Fig). The rank aggregation also indicated these two genes as most unstable (Fig 4). Notable is *Gapdh*, which comes out as the second-best normalizer, while in a KA intrahippocampal model this gene was found to be an inadequate reference [10]. Furthermore, related models mimicking the same disease could differ significantly in reference gene stabilities, since *Gusb* was an unstable gene in the systemic PILO model, but stable in the intrahippocampal PILO model [14].

In the end, we believe that the reference genes are less affected in the acute phase, except for *B2m* and *Gusb* which were significantly upregulated. The systemic KA administration does influence the global expression of some well-accepted reference genes (e.g. *Yhwaz* and *Hprt1*) and their stability decreases with progression of the disease, when aberrant mossy fiber sprouting is more prominent. The B1 element remains more or less stable in M values or stability values independent of brain region or disease stage. This study demonstrates the potential of implementing of the B1 element as a new reference for correct normalization of target genes in the systemic KA induced rat model of TLE. We are currently investigating whether its implementation as a normalization strategy can be expanded to other disease models or rodent species.

## Conclusion

Accurate normalization is essential in RT-qPCR studies to infer correct conclusions from the generated data. In this study, we investigated the stability of nine well-accepted reference genes and the transcripts of two SINEs (i.e. B1 and B2 element). The stability of the B elements and the performance as a normalizer were validated in a TLE rodent model induced by the systemic administration of KA. The exact same expression data resulted in different ranks between the geNorm and NormFinder algorithms. It is advisable to implement more than one algorithm in reference studies, or skewed conclusions may be made. The weighted rank aggregation generated a consensus list from both algorithms, but, importantly, this list was independent of brain region (i.e. hippocampus or cortex) and disease stage. Overall, the expression of the B1 element appears to be most stable, while *B2m* and *Gusb* were rather unreliable, even significantly upregulated in the acute phase of the TLE model in both evaluated brain regions. The validation of the new normalization strategy using *Gfap*, showed that the B1 element was comparable with the best pair of reference genes generated by geNorm and NormFinder. Thus, the B1 element can be implemented for the normalization of genes in this rodent model. Its general application in rodents should be studied further and confirmed by other pathology models and tissues.

## Supporting information

S1 Fig Relative expression analysis of *Gusb* and *B2m* in the hippocampus and the cortex. (A) The relative expression of *B2m* in the hippocampus. (B) The relative expression of *B2m* in the cortex. (C) The relative expression of *Gusb* in the hippocampus. (D) The relative expression of *Gusb* in the cortex. The graphs show the mean expression during the acute (control phase acute vs. T10) and chronic (control phase chronic vs. T80/T120) phase normalized by the optimal set of references genes described by geNorm, Mann-Whitney U Test or Kruskal-Wallis Test respectively, * = P ≤ 0.05, ** = P ≤ 0.01.
(TIFF)

S1 File R Script of the RankAggreg package. (PDF)

S1 Table Intra- and intervalues of the NormFinder results. (PDF)
