# Supplementary material for: The validation of Short Interspersed Nuclear Elements (SINEs) as a RT-qPCR normalization strategy in a rodent model for temporal lobe epilepsy
Source: PLoS One. 2019 Jan 10;14(1):e0210567. doi: 10.1371/journal.pone.0210567 (PMC6328105; doi:10.1371/journal.pone.0210567)
Supplement: S1 File — (PDF) [file pone.0210567.s002.pdf]

S1 File. R Script of the RankAggreg package.

```
> library(rstudioapi)
> library(RankAggreg)
> library(gtools)
> x2 = matrix(c("Pgk1", "Hprt1", "Tbp", "Ywhaz", "B1 Element", "Gapdh", "B2 Element", "Rpl13a", "Actb", "Gusb", "B2m",
+ 	"B2 Element", "B1 Element", "Gapdh", "Rpl13a", "Actb", "Pgk1", "Ywhaz", "Hprt1", "Tbp", "B2m", "Gusb",
+ 	"Hprt1", "Ywhaz", "Tbp", "Gapdh", "Pgk1", "Actb", "Rpl13a", "B2 Element", "B1 Element", "Gusb", "B2m",
+ 	"B1 Element", "B2 Element", "B2m", "Gapdh", "Actb", "Rpl13a", "Pgk1", "Tbp", "Gusb", "Hprt1", "Ywhaz",
+ 	"Rpl13a", "Actb", "Tbp", "B2 Element", "B1 Element", "Pgk1", "Gapdh", "Ywhaz", "Hprt1", "Gusb", "B2m",
+ 	"Rpl13a", "Gapdh", "B1 Element", "Hprt1", "Actb", "Pgk1", "Gusb", "Ywhaz", "B2 Element", "B2m", "Tbp",
+ 	"Actb", "Gapdh", "Pgk1", "Hprt1", "Ywhaz", "Tbp", "Rpl13a", "B2 Element", "B1 Element", "Gusb", "B2m",
+ 	"B2m", "B1 Element", "Tbp", "Pgk1", "Gapdh", "B2 Element", "Actb", "Gusb", "Hprt1", "Rpl13a", "Ywhaz"), byrow=TRUE,
ncol=11)

> w2 = matrix (c(.334, .258, .300, .474, .286, .415,  .181, .178, .317, .187, .205,
+ 	.256, .186, .173, .384, .197, .411, .309, .282, .225, .350, .295,
+ 	.175, .248, .229, .350, .138, .229, .095, .158, .200, .102, .099,
+ 	.370, .246, .256, .296, .339, .540, .587, .419, .388, .481, .648,
+ 	.185, .231, .193, .693, .334, .611, .381, .278, .164, .190, .337,
+ 	.229, .159, .298, .314, .143, .274, .211, .257, .129, .345, .280,
+ 	.112, .233, .228, .508, .145, .356, .163, .159, .216, .208, .168,
+ 	.266, .240, .264, .196, .261, .331, .333, .261, .359, .251, .437),
byrow=TRUE, ncol=11)

> w2 = t(apply(w2, 1, sort))

> (toplist2 = RankAggreg(x2, 11, w2, method="CE", distance="Spearman", N=1000, convIn=11, rho=.1)) # using the Spearman distance
```

The optimal list is:

B1 Element Gapdh Rpl13a Pgk1 Actb B2 Element Tbp Hprt1 Ywhaz B2m Gusb

Algorithm:	CE
Distance:	Spearman
Score:	8.852696
