# Supplementary material for: The validation of Short Interspersed Nuclear Elements (SINEs) as a RT-qPCR normalization strategy in a rodent model for temporal lobe epilepsy
Source: PLoS One. 2019 Jan 10;14(1):e0210567. doi: 10.1371/journal.pone.0210567 (PMC6328105; doi:10.1371/journal.pone.0210567)
Supplement: S1 Table — (PDF) [file pone.0210567.s003.pdf]

S1 Table. Intra- and intervalues of the NormFinder results.

Gene name	Intragroup Acute Phase Hippocampus		Intergroup Acute Phase Hippocampus	
	Control	T10	Control	T10
Actb	0.051	0.153	0.012	-0.012
B1 Element	0.041	0.051	-0.113	0.113
B2 Element	0.018	0.154	-0.048	0.048
B2m	0.051	0.002	0.632	-0.632
Gapdh	0.014	0.160	-0.203	0.203
Gusb	0.036	0.013	0.536	-0.536
Hprt1	0.015	0.049	-0.291	0.291
Pgk1	0.009	0.004	-0.234	0.234
Rpl13a	0.071	0.068	0.015	-0.015
Tbp	0.048	0.003	-0.114	0.114
Ywhaz	0.051	0.111	-0.192	0.192

Gene name	Intragroup Chronic Phase Hippocampus			Intergroup Chronic Phase Hippocampus		
	Control	T80	T120	Control	T80	T120
Actb	0.068	0.005	0.108	-0.232	0.047	0.185
B1 Element	0.105	0.021	0.046	0.017	0.051	-0.068
B2 Element	0.035	0.198	0.118	0.276	-0.033	-0.243
B2m	0.019	0.266	0.167	0.297	-0.127	-0.170
Gapdh	0.018	0.035	0.035	0.085	-0.082	-0.003
Gusb	0.040	0.115	0.368	0.196	0.028	-0.223
Hprt1	0.029	0.055	0.089	-0.155	0.149	0.006
Pgk1	0.014	0.066	0.068	-0.255	0.126	0.129
Rpl13a	0.034	0.020	0.004	-0.096	0.026	0.070
Tbp	0.313	0.079	0.227	0.174	-0.335	0.160
Ywhaz	0.002	0.116	0.045	-0.306	0.149	0.157

Gene name	Intragroup Acute Phase Cortex		Intergroup Acute Phase Cortex	
	Control	T10	Control	T10
Actb	0.026	0.008	0.043	-0.043
B1 Element	0.033	0.064	-0.128	0.128
B2 Element	0.283	0.110	0.005	-0.005
B2m	0.033	0.069	0.439	-0.439
Gapdh	0.017	0.022	-0.070	0.070
Gusb	0.126	0.105	0.232	-0.232
Hprt1	0.003	0.013	-0.117	0.117
Pgk1	0.023	0.065	-0.051	0.051
Rpl13a	0.037	0.050	-0.112	0.112
Tbp	0.020	0.025	-0.131	0.131
Ywhaz	0.006	0.020	-0.110	0.110

Gene name	Intragroup Chronic Phase Cortex			Intergroup Chronic Phase Cortex		
	Control	T80	T120	Control	T80	T120
Actb	0.019	0.106	0.124	-0.245	0.257	-0.012
B1 Element	0.223	0.171	0.151	-0.092	0.114	-0.022
B2 Element	0.397	0.154	0.209	-0.120	0.113	0.007
B2m	0.076	0.032	0.098	0.088	0.055	-0.143
Gapdh	0.042	0.132	0.150	-0.227	0.219	0.008
Gusb	0.588	0.092	0.420	0.088	-0.229	0.141
Hprt1	0.677	0.287	0.305	0.247	-0.230	-0.017
Pgk1	0.014	0.030	0.074	0.216	0.092	-0.308
Rpl13a	0.132	0.356	0.501	-0.378	0.257	0.120
Tbp	0.119	0.195	0.270	0.053	-0.143	0.090
Ywhaz	0.633	0.738	0.916	0.369	-0.506	0.136
